# Longitudinal analysis of the vaginal microflora in pregnancy suggests that *L. crispatus *promotes the stability of the normal vaginal microflora and that *L. gasseri *and/or *L. iners *are more conducive to the occurrence of abnormal vaginal microflora

**DOI:** 10.1186/1471-2180-9-116

**Published:** 2009-06-02

**Authors:** Hans Verstraelen, Rita Verhelst, Geert Claeys, Ellen De Backer, Marleen Temmerman, Mario Vaneechoutte

**Affiliations:** 1 Department of Obstetrics & Gynaecology, Faculty of Medicine and Health Sciences, Ghent University, Ghent, Belgium; 2 Laboratory Bacteriology Research, Department of Clinical Chemistry, Microbiology and Immunology, Faculty of Medicine and Health Sciences, Ghent University, Ghent, Belgium; 3 Department of Clinical Chemistry, Microbiology, and Immunology, Faculty of Medicine and Health Sciences, Ghent University, Ghent, Belgium

## Abstract

**Background:**

Despite their antimicrobial potential, vaginal lactobacilli often fail to retain dominance, resulting in overgrowth of the vagina by other bacteria, as observed with bacterial vaginosis. It remains elusive however to what extent interindividual differences in vaginal *Lactobacillus *community composition determine the stability of this microflora. In a prospective cohort of pregnant women we studied the stability of the normal vaginal microflora (assessed on Gram stain) as a function of the presence of the vaginal *Lactobacillus *index species (determined through culture and molecular analysis with tRFLP).

**Results:**

From 100 consecutive Caucasian women vaginal swabs were obtained at mean gestational ages of 8.6 (SD 1.4), 21.2 (SD 1.3), and 32.4 (SD 1.7) weeks, respectively. Based on Gram stain, 77 women had normal or *Lactobacillus*-dominated vaginal microflora (VMF) during the first trimester, of which 18 had grade Ia (*L. crispatus *cell morphotypes) VMF (23.4%), 16 grade Iab (*L. crispatus *and other *Lactobacillus *cell morphotypes) VMF (20.8%), and 43 grade Ib (non-*L. crispatus *cell morphotypes) VMF (55.8%). Thirteen women with normal VMF at baseline, converted in the second or third trimester (16.9%) to abnormal VMF defined as VMF dominated by non-*Lactobacillus *bacteria. Compared to grade Ia and grade Iab VMF, grade Ib VMF were 10 times (RR = 9.49, 95% CI 1.30 – 69.40) more likely to convert from normal to abnormal VMF (p = 0.009). This was explained by the observation that normal VMF comprising *L. gasseri/iners *incurred a ten-fold increased risk of conversion to abnormal VMF relative to non-*L. gasseri/iners *VMF (RR 10.41, 95% CI 1.39–78.12, p = 0.008), whereas normal VMF comprising *L. crispatus *had a five-fold decreased risk of conversion to abnormal VMF relative to non-*L. crispatus *VMF (RR 0.20, 95% CI 0.05–0.89, p = 0.04).

**Conclusion:**

The presence of different *Lactobacillus *species with the normal vaginal microflora is a major determinant to the stability of this microflora in pregnancy: *L. crispatus *promotes the stability of the normal vaginal microflora while *L. gasseri *and/or *L. iners *predispose to some extent to the occurrence of abnormal vaginal microflora.

## Background

Under normal conditions, the lower female genital tract harbours a mutualistic microflora that primarily consists of lactobacilli which confer antimicrobial protection to the vagina as a critical port of entry for local, ascending and systemic infectious disease [1,2]. The lactobacilli-driven defence of the vaginal niche is in its essence seized as a principle of colonisation resistance, i.e. the vaginal lactobacilli prevent colonisation of the vaginal epithelium by other microorganisms, through a variety of mechanisms [3].

Despite their intrinsic antimicrobial potential however, vaginal lactobacilli fail to retain dominance in a considerable number of women, resulting in overgrowth of the vaginal epithelium by other bacteria, as observed, most typically, with anaerobic polymicrobial overgrowth in bacterial vaginosis [1], or less commonly, with overgrowth by streptococci, including group A [4] and group B streptococci [5,6], by bifidobacteria [7,8], or by coliforms such as *E. coli *[5,6,9].

Loss of the indigenous lactobacilli strongly predisposes to ascending genital tract infection, which in pregnancy is a major cause of chorioamnionitis, amniotic fluid infection, and preterm birth [1,2]. A depletion of the vaginal *Lactobacillus *microflora further predisposes to the acquisition of sexually transmitted infectious diseases such as gonorrhoea [10,11], chlamydiosis [11], and HIV infection [12,13].

The mechanisms involved in the loss of the mutualistic lactobacilli remain largely unknown and hence it remains elusive whether lactobacilli for some reason are losing ground thereby allowing other microorganisms to proliferate or whether other bacteria for some reason elicit overgrowth thereby displacing the resident lactobacilli. In fact, the ecological conditions of the vaginal niche, like the low pH, which enable lactobacilli to inhibit overgrowth by other microorganisms, are intermittently disturbed by non-microbial factors, primarily by the menses [14] and by sexual intercourse [15], to which some practices like vaginal douching [16] may add further disturbance.

It has not been extensively investigated however to what extent interindividual differences in vaginal *Lactobacillus *community composition determine the stability of this microflora neither how differences in host innate immunity contribute to interindividual differences in susceptibility to bacterial overgrowth of the vagina. The normal vaginal microflora has recently been found to consist primarily of one or more of merely four distinct species, in particular *L. crispatus, L. jensenii*, *L. gasseri *and *L. iners *[7,17,18]. Here, we established the stability of the vaginal microflora during pregnancy as a function of the presence of each of these index species, in a prospective cohort study.

## Results

From 100 consecutive Caucasian women vaginal swabs for Gram stain-based microscopy, tRFLP, and culture were obtained at mean gestational ages of 8.6 (SD 1.4), 21.2 (SD 1.3), and 32.4 (SD 1.7) weeks, respectively.

### Vaginal microflora status according to Gram stain at baseline and on follow-up

Based on Gram stain, 77 women presented with normal or grade I vaginal microflora (VMF) during the first trimester, of which 18 had grade Ia (primarily *L. crispatus *cell morphotypes) VMF (23.4%), 16 grade Iab (*L. crispatus *and other *Lactobacillus *cell morphotypes) VMF (20.8%), and 43 grade Ib (primarily non-*L. crispatus *cell morphotypes) VMF (55.8%). Of these, 64 women (83.1%) maintained grade I VMF throughout pregnancy, whereas 13 women with grade I VMF during the first trimester, converted to abnormal VMF in the second or third trimester (16.9%) (Table 1). Conversely, of the 23 women with abnormal VMF in the first trimester (grade I-like (5), grade II (11), grade III (4), and grade IV (3)), 13 reconverted to normal VMF (56.5%) in the second or third trimester (Table 2).

**Table 1:** Overview of microflora patterns for patients who displayed a conversion from normal to abnormal microflora (n = 13)

	Microflora grade on Gram stain		
patient number	trimester I	trimester II	trimester III

*PB2003/003*	Ib	I-like	I-like

*PB2003/007*	Ib	III	Ia

*PB2003/013*	Ib	II	Ib

*PB2003/018*	Ia	Ia	I-like

*PB2003/019*	Ib	II	II

*PB2003/049*	Ib	Ib	II

*PB2003/084*	Ib	II	Ia

*PB2003/101*	Iab	Ib	II

*PB2003/116*	Ib	I-like	II

*PB2003/130*	Ib	I-like	Ib

*PB2003/147*	Ib	Ib	I-like

*PB2003/148*	Ib	Ib	II

*PB2003/155*	Ib	Ib	II

Gram stained vaginal smears were scored according to the criteria previously described by Verhelst *et al *[7]. Briefly, Gram-stained vaginal smears were categorized as grade I (normal) when only *Lactobacillus *cell types were present, as grade II (intermediate) when both *Lactobacillus *and bacterial vaginosis-associated cell types were present, as grade III (bacterial vaginosis) when bacterial vaginosis-associated cell types were abundant in the absence of lactobacilli, as grade IV when only gram-positive cocci were observed, and as grade I-like when irregularly shaped or curved gram-positive rods were predominant [7]. For the purpose of this study, grade I or *Lactobacillus*-dominated vaginal microflora is designated as 'normal vaginal microflora' and all other grades as 'abnormal vaginal microflora'.

**Table 2:** Overview of microflora patterns on Gram stain on follow-up for patients who displayed an abnormal microflora in the first trimester (n = 23)

patient number	trimester I	trimester II	trimester III
*PB2003/070*	I-like	Ib	Ia

*PB2003/106*	I-like	Ib	Ib

*PB2003/120*	I-like	III	Ia

*PB2003/117*	I-like	I-like	I-like

*PB2003/088*	I-like	I-like	IV

*PB2003/121*	II	Ia	Ia

*PB2003/123*	II	Iab	Ia

*PB2003/012*	II	Ib	Ib

*PB2003/108*	II	I-like	Ia

*PB2003/063*	II	I-like	I-like

*PB2003/076*	II	II	Ib

*PB2003/017*	II	III	Ib

*PB2003/080*	II	I-like	IV

*PB2003/044*	II	II	I-like

*PB2003/046*	II	II	II

*PB2003/105*	II	II	II

*PB2003/078*	III	Ib	Ib

*PB2003/079*	III	Ib	Ib

*PB2003/094*	III	I-like	Ia

*PB2003/132*	III	III	III

*PB2003/144*	IV	I-like	Ib

*PB2003/025*	IV	I-like	I-like

*PB2003/008*	IV	IV	IV

Gram stained vaginal smears were scored according to the criteria previously described by Verhelst *et al *[7]. Briefly, Gram-stained vaginal smears were categorized as grade I (normal) when only *Lactobacillus *cell types were present, as grade II (intermediate) when both *Lactobacillus *and bacterial vaginosis-associated cell types were present, as grade III (bacterial vaginosis) when bacterial vaginosis-associated cell types were abundant in the absence of lactobacilli, as grade IV when only gram-positive cocci were observed, and as grade I-like when irregularly shaped or curved gram-positive rods were predominant [7]. For the purpose of this study, grade I or *Lactobacillus*-dominated vaginal microflora is designated as 'normal vaginal microflora' and all other grades as 'abnormal vaginal microflora'.

Among the 13 women with grade I VMF during the first trimester and who converted in the second or third trimester to abnormal VMF (Table 1), the transition involved once a transition from grade Ia VMF to abnormal VMF (grade I-like) (1/18 or 5.6%), twelve times a transition from grade Ib VMF to abnormal VMF (grade I-like (4), grade II (7), and grade III (1)) (12/43 or 27.9%), while none of the 16 women with grade Iab VMF converted to abnormal VMF (Table 1). Accordingly, compared to grade Ia and grade Iab VMF, grade Ib VMF were about 10 times (RR = 9.49, 95% CI 1.30 – 69.40) more likely to convert from normal to abnormal VMF (p = 0.009).

### Prevalence of Lactobacillus species according to tRFLP and culture at baseline and on follow-up

We further elaborated on the above findings through the study of the prevalence over time of the distinct *Lactobacillus *species as determined through tRFLP and culture. Through tRFLP and culture, the vaginal lactobacilli comprising the grade I VMF were identified to be predominantly one or more of four different *Lactobacillus *species, i.e., *L. crispatus*, *L. jensenii*, *L. gasseri *and *L. iners *(Table 3). As the latter two species could not be differentiated from each other on tRFLP analysis and since both species could not be cultured in 9 cases, their presence is further referred to as *L. gasseri*/*L. iners*.

**Table 3:** Composition of grade I microbiota according to culture and tRFLP in the first pregnancy trimester (n = 77)

*L. crispatus (only)*	23.4% (18)
*L. jensenii *(only)	3.9% (3)
*L. gasseri/L. iners *(only)	40.3% (31)
	
*L. crispatus *+ *L. jensenii*	15.6% (12)
*L. crispatus *+ *L. gasseri/L. iners*	9.1% (7)
*L. jensenii *+ *L. gasseri/L. iners*	3.9% (3)
*L. crispatus *+ *L. jensenii *+ *L. gasseri/L. iners*	2.6% (2)
	
*unidentified*	*1.3% (1)*

*L. crispatus*,*L. jensenii*, and *L. gasseri/iners *were *present with 39, 20, and 43 women in the first trimester respectively*. When accounting for the entire follow-up period, *L. crispatus *persisted at a rate of 92.3%, *L. jensenii *at a rate of 80.0% and *L. gasseri/iners *at a rate 69.8% (Table 4).

**Table 4:** Overview of the prevalence of the *Lactobacillus *index species at three consecutive points in time during pregnancy for the 77 women with grade I microflora during the first trimester

*Lactobacillus *species as determined through culture and tRFLP (N = 77)			
	trimester I (n)	trimester II (n)	trimester III (n)

all samples with an *L. crispatus *TRF	39 (100%)	37 (94.9%)	36 (92.3%)
all samples with an *L. jensenii *TRF	20 (100%)	18 (90.0%)	16 (80.0%)
all samples with an *L. gasseri/iners *TRF	43 (100%)	36 (83.7%)	30 (69.8%)

We subsequently accounted for changes in the prevalence of *Lactobacillus *index species by accounting for the first-to-second and second-to-third trimester transitions respectively.

*L. crispatus *was present in 39 respectively 44 women with grade I VMF during the first respectively second trimester. When accounting for the first-to-second and second-to-third trimester transitions respectively, *L. crispatus *disappeared twice (5.1%) respectively once (2.3%). So, overall, *L. crispatus *as a member of the normal VMF (n = 83) persisted in the vast majority of cases (96.4%) throughout the following trimester.

*L. jensenii *in turn was present in 20 respectively 22 women with grade I VMF during the first respectively second trimester. When accounting for the first-to-second and second-to-third trimester conversions respectively, *L. jensenii *disappeared on two (10.0%) respectively five occasions (22.7%). So, overall, *L. jensenii *occurring with normal VMF (n = 42), sustained throughout a subsequent trimester at a rate of 83.3%. Hence, *L. jensenii *was found to be a significantly less stable microflora component as compared to *L. crispatus*, with the likelihood of *L. jensenii *disappearing equalling a McNemar odds ratio of 11.67 (95% CI 3.45 – 47.51, p < 0.001).

*L. gasseri *and/or *L. iners *– designated *L. gasseri/iners *– were present in 43 respectively 40 women with grade I VMF during the first respectively second trimester. When accounting for the first-to-second and second-to-third trimester conversions, *L. gasseri/iners *got lost on seven (16.3%) respectively six occasions (15.0%). Accordingly, *L. gasseri/iners *as members of the normal VMF (n = 83) continued to be present in a following trimester at a rate of 84.3%. Hence, compared to *L. crispatus*, *L. gasseri *and/or *iners *were found to be significantly less stable microflora components (McNemar odds ratio 23.33, 95% CI 7.10 – 92.69, p < 0.001).

### Association between the presence of distinct Lactobacillus species at baseline and vaginal microflora status on follow-up

We explored whether the observations on the stability of the grade I VMF as determined by Gram stain correlated with the observations on the stability of the distinct *Lactobacillus *species observed with grade I VMF as determined through tRFLP and culture.

Normal microflora comprising *L. crispatus *as a member (n = 83) rarely shifted away from grade I VMF microflora (2.4%). Such a shift was observed on merely two occasions (shift to grade I-like and grade II VMF respectively) and was not associated with the disappearance of *L. crispatus *(Table 5). Normal microflora comprising *L. jensenii *as a member (n = 42) shifted away from grade I VMF microflora on three occasions (on each occasion involving a grade Ib VMF to grade II VMF transition) (7.2%), and on two occasions this was associated with the loss of *L. jensenii *(Table 5). Finally, normal VMF comprising *L. gasseri/iners *as a member (n = 83) converted to abnormal VMF on twelve occasions (involving conversion from grade I VMF to grade I-like five times, to grade II six times, and to grade III once) (14.5%) (Table 5), which was associated with the disappearance of *L. gasseri/iners *in merely two out of the twelve normal to abnormal VMF transitions. It may be added that in the aforementioned instances, including the two *L. crispatus *comprising VMF and the three *L. jensenii *comprising VMF which converted to abnormal VMF, *L. gasseri/iners *was actually present alongside *L. crispatus *respectively *L. jensenii*. So, in summary conversion from normal VMF to abnormal VMF was associated twice with grade I *L. crispatus *+ *L. gasseri/iners *microflora, three times with grade I *L. jensenii *+ *L. gasseri/iners *microbiota, and seven times with grade I microbiota only containing *L. gasseri/iners*. In one additional case, conversion from normal VMF to abnormal VMF occurred with a woman with grade Ib VMF from which no lactobacilli could be identified through tRFLP and culture.

**Table 5:** Association between *Lactobacillus *type as part of grade I microflora (on culture and tRFLP) and microflora status (on Gram stain) on follow-up when accounting for the first-to-second and second-to-third trimester transitions

*Lactobacillus *species (culture and tRFLP) at baseline	Gram stain category on follow-up
all samples with an *L. crispatus *TRF (n = 83)	
▪ sustained grade I microflora	97.6% (81)
▪ shift to an abnormal microflora	
- grade I-like	1.2% (1)
- grade II	1.2% (1)
- grade III	-
- grade IV	-
all samples with an *L. jensenii *TRF (n = 42)	
▪ sustained grade I microflora	92.9% (39)
▪ shift to an abnormal microflora	
- grade I-like	-
- grade II	7.1% (3)
- grade III	-
- grade IV	-
all samples with an *L. gasseri/iners *TRF (n = 83)	
▪ sustained grade I microflora	85.5% (71)
▪ shift to an abnormal microflora	
- grade I-like	6.0% (5)
- grade II	7.2% (6)
- grade III	1.2% (1)
- grade IV	-

Gram stained vaginal smears were scored according to the criteria previously described by Verhelst *et al *[7]. Briefly, Gram-stained vaginal smears were categorized as grade I (normal) when only *Lactobacillus *cell types were present, as grade II (intermediate) when both *Lactobacillus *and bacterial vaginosis-associated cell types were present, as grade III (bacterial vaginosis) when bacterial vaginosis-associated cell types were abundant in the absence of lactobacilli, as grade IV when only gram-positive cocci were observed, and as grade I-like when irregularly shaped or curved gram-positive rods were predominant [7]. For the purpose of this study, grade I or *Lactobacillus*-dominated vaginal microflora is designated as 'normal vaginal microflora' and all other grades as 'abnormal vaginal microflora'.

### Summary of the association between normal microflora type and vaginal microflora status on follow-up

Overall, in this cohort, normal VMF at baseline examination shifted to an abnormal VMF on follow-up at a rate of 16.9%, whereby – according to Gram stain – 92.3% of these cases were associated with a departure from grade Ib VMF and – according to tRFLP and culture – 92.3% of these cases involved a departure from grade I VMF comprising *L. gasseri/iners*. Conversely, the presence of *L. crispatus *even when accompanied by the other Lactobacillus species, *L. jensenii*, *L. gasseri and/or L. iners*, emerged as a prominent stabilising factor to the vaginal microflora. In particular, normal VMF comprising *L. gasseri/iners *incurred a ten-fold increased risk of conversion to abnormal VMF relative to non-*L. gasseri/iners *VMF (RR 10.41, 95% CI 1.39–78.12, p = 0.008), whereas normal VMF comprising *L. crispatus *had a five-fold decreased risk of conversion to abnormal VMF relative to non-*L. crispatus *VMF (RR 0.20, 95% CI 0.05–0.89, p = 0.04).

Of importance is that, while on the one hand it was observed that *L. jensenii *and *L. gasseri/iners *tended to disappear at a significantly higher rate over time (i.e. displaying poorer colonisation strength) as compared to *L. crispatus*, and on the other hand that *L. jensenii *and in particular *L. gasseri/iners *were associated with a much higher risk of conversion from normal to abnormal VMF (i.e. displaying poorer colonisation resistance), these phenomena did not seem to be interrelated, i.e. conversion to abnormal VMF is mostly accompanied by the persistence rather than the disappearance of the *Lactobacillus *index species. Hence, it appears as if *L. jensenii *and *L. gasseri/iners *in particular, elicit in comparison to *L. crispatus *both poorer colonisation strength and poorer colonisation resistance.

### Longitudinal analysis of the prevalence of Lactobacillus species according to culture and tRFLP with advancing pregnancy

Finally, we examined the trends in the occurrence of the distinct *Lactobacillus *species as indentified through culture and tRFLP with advancing pregnancy. When accounting for the subsequent trimesters *L. crispatus *was present in 42, 49, and 60 of the 100 women respectively, *L. jensenii *in 27, 33, and 32 patients, and *L. gasseri/iners *in 59, 57, and 49 subjects, respectively. Accordingly, there was a significant positive trend in the occurrence of *L. crispatus *(χ^2 ^test-for-trend = 6.46, p = 0.011), while there was no significant trend in the prevalence of *L. jensenii *(χ^2 ^test-for-trend = 0.59, p = 0.4), nor in the occurrence of *L. gasseri/iners *(χ^2 ^test-for-trend = 2.01, p = 0.2). Hence a significant increase in the presence of *L. crispatus *with grade I VMF (prevalence ratio 1.32, 95% CI 1.01 – 1.72, p = 0.04) from the first to the third trimester was observed, whereas conversely there was a trend towards a decreased presence of *L. gasseri/iners *with grade I VMF (prevalence ratio 0.77, 95% CI 0.56 – 1.06, p = 0.1), albeit non-significant. Consequently while there was no significant trend in the prevalence of normal VMF with advancing pregnancy in this cohort, a larger number of women with normal VMF gained *L. crispatus*.

## Discussion

The vaginal lactobacilli were originally described in the late 19^th ^century by German gynaecologist Albert Döderlein, who purported that the lactobacilli act as a barrier of defence preventing other bacteria to ascend the genital tract [19]. Since then, it has been established that the vaginal lactobacilli are indeed capable of providing colonisation resistance through a variety of mechanisms. Nonetheless, failure of the lactobacilli-driven defence often occurs, resulting in overgrowth of the vaginal epithelium by other bacteria, as observed, most typically, with anaerobic polymicrobial overgrowth in bacterial vaginosis and less commonly with overgrowth by bifidobacteria [7,8] and other bacteria.

From this perspective, major interest in the study of the vaginal lactobacilli has emerged in recent years, as it is assumed that thorough characterisation of the normal vaginal microflora may provide us with a better understanding of the mechanisms involved with the stability of lactobacilli-dominated microflora, or conversely, with their failure to maintain the vaginal ecosystem. It was recently established that of the 80 known *Lactobacillus *species, up to 20 different species may colonize the intestinal tract, yet merely four species seem to dominate the vaginal microflora, in particular *L. crispatus*, *L. jensenii, L. gasseri *and *L. iners *[7,17,18], a finding that has now been corroborated in various parts of the world among women with differing ethnicity[20], albeit a fifth species *L. vaginalis *may have been overlooked by culture-independent methods (unpublished data). Based on the distinctive morphology of the *Lactobacillus *index species, we previously showed that it is possible to differentiate between various types of normal or grade I vaginal microflora, in particular grade Ia when only *Lactobacillus crispatus *cell types (plump, mostly short rods) are present, grade Ib when only other *Lactobacillus *cell types are present (smaller or more elongated and less stained than in Ia smears) and as grade Iab when both *L. crispatus *and other lactobacilli are present [7].

In the present study it could be shown that of all women who presented with normal or grade I VMF during the first trimester and who converted to abnormal VMF in the second or third trimester, the shift from normal to abnormal VMF was for the most part preceded by the presence of grade Ib VMF, whereas grade Ia and Iab VMF rarely shifted away to an abnormal VMF. We further explored whether this finding translated to the *Lactobacillus *species level through culture and tRFLP fingerprinting. It could be shown that grade I VMF comprising *L. crispatus *shifted away to abnormal VMF in merely 2.4% of the cases, whereas grade I VMF *containing L. gasseri/iners *converted to abnormal VMF at a rate of 14.5% of the cases respectively. Accordingly, normal VMF comprising *L. gasseri/iners *incurred a ten-fold increased risk of conversion to abnormal VMF relative to non-*L. gasseri/iners *VMF (RR 10.41, 95% CI 1.39–78.12, p = 0.008), whereas normal VMF comprising *L. crispatus *had a five-fold decreased risk of conversion to abnormal VMF relative to non-*L. crispatus *VMF (RR 0.20, 95% CI 0.05–0.89, p = 0.04).

The observation that *L. gasseri/iners *comprising VMF apparently offers significantly less stability as compared to *L. crispatus *containing VMF, was not explained however by the higher rate at which *L. gasseri/iners *disappeared on follow-up, or hence by their lower colonisation strength. Rather it appears as if *L. gasseri *and *L. iners *offer poorer colonisation resistance thereby allowing the overgrowth of other bacteria. This finding concurs at least in part with what we recently reported, i.e., contrary to the traditional contention that the progression of normal over intermediate to bacterial vaginosis VMF involves the disappearance of the vaginal lactobacilli, we showed that *L. gasseri *proliferates with intermediate VMF and that *L. iners *growth is enhanced with bacterial vaginosis [21].

Hence, from the present study on the natural history of the normal vaginal microflora in pregnant women, it appears that *L. crispatus*, is associated with a particularly stable vaginal ecosystem. Conversely, microflora comprising *L. jensenii *elicits intermediate stability, while VMF comprising *L. gasseri/L. iners *is the least stable.

Interestingly, Kalra *et al *recently suggested that bacterial vaginosis might arise selectively from subtypes of normal microflora and that recolonisation with *L. iners *following an episode of bacterial vaginosis might be a risk factor for recurrence [22]. Ferris *et al *analysed the vaginal microflora among six patients treated with 0.75% topical metronidazole gel applied once daily for five days and found at 30 days posttreatment that a single species, *L. iners*, was predominant in all patients, except for the one patient for whom treatment failed both according to Nugent and Amsel criteria [23]. Hence, it has been suggested that following the resolution of bacterial vaginosis, *L. iners *is the only *Lactobacillus *species that succeeds to replenish the vagina in appreciable amounts, which in turn may render these patients more vulnerable to a new episode of bacterial vaginosis, considering the rather moderate colonisation resistance offered by *L. iners *[22]. Jakobsson and Forsum corroborated the finding by Ferris *et al *and further suggested that *L. iners *may become a dominant part of the vaginal microflora when the microflora is in a transitional stage between abnormal and normal [24].

As our study was confined to genotypic characterisation of the microflora, it remains to be determined which phenotypic attributes of the different *Lactobacillus *species explain the observed associations. Previous studies have pointed at an important role for hydrogen peroxide production in colonization resistance [25-27]. In a 2-year follow-up study, Hawes *et al *documented that the acquisition of bacterial vaginosis was strongly associated with a lack or loss of hydrogen peroxide producing lactobacilli [28]. At first sight, our findings corroborate this paradigm, as most *L. crispatus *strains have been found to be very consistently strong H_2_O_2 _producers [29,30], whereas most *L. iners *strains have been found to be for the most part non-H_2_O_2 _producers [29,30]. However, other factors must be involved as well. In particular, most *L. jensenii *strains have been found to be equally strong H_2_O_2 _producers as *L. crispatus *[29,30], although in this study *L. jensenii *showed a stronger association with conversion to abnormal VMF. A possible explanation is that *L. jensenii *is the only *Lactobacillus *species for which poorer colonisation resistance seemed to be correlated with poorer colonisation strength, i.e. conversion to abnormal VMF was more likely to be associated with the disappearance of *L. jensenii*. Compared to the other *Lactobacillus *species, *L. jensenii *is also on average present in a significantly lower concentration with grade I VMF [21].

Our results must be taken with extreme caution as our study had several important limitations. Firstly, our sample size was rather small and therefore our results need to be corroborated in larger cohorts. Secondly, we acknowledge that the interval between subsequent sampling occasions was rather large with an average of some 3 months interval time. Thirdly, it must be acknowledged that a single sampling occasion may not properly reflect the vaginal microflora status of a woman due to swift changes in the microflora as has been documented previously [31,32]. Fourthly, due to the choice of the *Bst*UI restriction enzyme, which generates equal-sized terminal fragment lengths for *L. gasseri *and *L. iners *on tRFLP, we were unable to differentiate between *L. gasseri *and *L. iners*, also because we failed to culture these species consistently. Previous studies have applied tRFLP more successfully in this respect [33,34]. Fifthly, it must be acknowledged that we did not record any behavioural factors that might have impinged on vaginal microflora status during the study. Though not necessarily confounding our results, this might have been most informative. Finally, as the study was conducted among pregnant women our results may not be representative for the natural history of the vaginal microflora among non-pregnant women.

## Conclusion

In conclusion, we believe to have documented that the presence of different *Lactobacillus *species with the normal vaginal microflora (VMF) is a major determinant to the stability of the VMF in pregnancy. The presence of *L. crispatus *seems to ensure ongoing presence of *L. crispatus *and normal VMF; the presence of *L. jensenii *is associated with normal VMF, but *L. jensenii *is more likely to disappear over time which may lead to overgrowth by other bacteria; the presence of *L. gasseri*/*L. iners *is likely to vary over time and strongly predisposes to bacterial overgrowth of the vagina in pregnancy. These observations are paramount in view of the vast disease burden associated with depleted lactobacilli and bacterial vaginosis in particular, a condition that affects at any time some 10 to 50% of women worldwide [35]. As a matter of fact, the whole point of it is that these observations appear to challenge the century-old paradigm of "normal" or "healthy" vaginal microflora, a state that is still defined by the enumeration of bacterial cell morphotypes on microscopy. In particular, as we found that some half of women actually have a microflora characterized by the poorer colonizers and defenders *L. gasseri *and *L. iners*, it may be inferred that in a substantial proportion of women lactobacilli-driven antimicrobial defence of the lower female genital tract is actually less optimal than can be assumed by the mere presence of lactobacilli.

## Methods

### Study Population

In a prospective cohort study, unselected pregnant women were consecutively enrolled through informed consent on the occasion of their first antenatal visit, from January 2003 through May 2004, at the outpatient obstetric clinic of the Ghent University Hospital [8]. Patients were scheduled to provide three vaginal swabs at three points in time corresponding to subsequent pregnancy trimesters. The first 100 women with complete series of swabs were considered for longitudinal analysis of the normal vaginal microflora.

### Clinical procedures

A cotton-tipped wooden vaginal swab (Euron, Ontex, Belgium) was rolled against the lateral vaginal walls, carefully withdrawn, and rolled out on a plain glass slide; the air-dried vaginal smear was then Gram-stained. A second, sterile cotton-tipped wooden swab (Sterilin, Copan, Italy) was rolled against the lateral vaginal walls and placed in a sterile polypropylene tube for transport. A third swab was obtained in a similar manner and placed into Amies transport medium (Nuova Aptaca, Canelli, Italy) for anaerobic culture.

### Grading of Gram-stained vaginal smears

The Gram stained vaginal smears were scored by two independent assessors (GC and RV) according to the criteria previously described by Verhelst *et al *[7]. Briefly, Gram-stained vaginal smears were categorized as grade I (normal) when only *Lactobacillus *cell types were present, as grade II (intermediate) when both *Lactobacillus *and bacterial vaginosis-associated cell types were present, as grade III (bacterial vaginosis) when bacterial vaginosis-associated cell types were abundant in the absence of lactobacilli, as grade IV when only gram-positive cocci were observed, and as grade I-like when irregularly shaped or curved gram-positive rods were predominant [7]. For the purpose of this study, grade I or *Lactobacillus*-dominated vaginal microflora is designated as 'normal vaginal microflora' and all other grades as 'abnormal vaginal microflora'.

### Culture and identification of cultured isolates by tDNA-PCR

The swab on Amies transport medium was streaked onto Schaedler agar enriched with 5% sheep blood, vitamin K, haemin and sodium pyruvate (Becton Dickinson, Franklin Lakes, NJ) and incubated anaerobically at 37°C upon arrival at the microbiology laboratory. After 4 days of incubation, all the isolates with different colony morphology were selected for identification. DNA was extracted by simple alkaline lysis: one colony was suspended in 20 μl of 0.25% sodium dodecyl sulfate-0.05 N NaOH, heated at 95°C for 15 min and diluted with 180 μl of distilled water. tDNA-PCR and capillary electrophoresis were carried out as described previously [36,37]. The species to which each isolate belonged was determined by comparing the tDNA-PCR fingerprint obtained from each isolate with a library of tDNA-PCR fingerprints obtained from reference strains, using an in-house software program [37]. The library of tDNA-PCR fingerprints is available at our website and the software can be obtained upon request [38].

### DNA extraction of vaginal swab samples

For DNA extraction from the dry vaginal swabs, the QIAamp DNA mini kit (Qiagen, Hilden, Germany) was used according to the manufacturer's recommendations, with minor modifications. The dry swab specimen from each patient was swirled for 15 s in 400 μl of lysis buffer (20 mM Tris-HCl, pH 8.0; 2 mM EDTA; 1.2% Triton). Fifty units of mutanolysin (25 U/μl) (Sigma, Bornem, Belgium) were added and the samples were incubated for 30 min at 37°C. After the addition of 20 μl Proteinase K (20 mg/ml) and 200 μl AL buffer (Qiagen), samples were incubated for 30 min at 56°C. Next, 200 μl of ethanol was added and DNA was purified by adding the lysate to the Qiagen columns as described by the manufacturer. Finally, the total bacterial DNA was eluted with 100 μl of AE buffer (Qiagen). DNA extracts were stored at -20°C and were used for the purpose of T-RFLP analysis and species specific PCR.

### tRFLP analysis

The forward primers 10f (5' TET-AGTTTGATCCTGGCTCAG) or GV10f (5' TET-GGTTCGATTCTGGCTCAG) and the reverse primer 534r (5' ATTACCGCGGCTGCTGG) [7,33] which target the 16S rRNA gene of the domain *Bacteria*, were used to amplify part of the 16S rDNA by PCR. Two 15 μl PCR mixtures contained respectively primer set 10f-534r or GV10f-534r at a final concentration of 0.1 μM of each primer and at a ratio of labelled and unlabelled forward primer of 2/3, 7.5 μl of Promega master mix (Promega, Madison, WI) 1.5 μl of sample and 5.9 μl HPLC water. Thermal cycling consisted of an initial denaturation of 5 min at 94°C, followed by three cycles of 1 min at 94°C, 2 min at 50°C and 1 min at 72°C, followed by 35 cycles of 20 sec at 94°C, 1 min at 50°C and 1 min 72°C, with a final extension of 10 min at 72°C, and cooling to 10°C. A 20 μl restriction mixture, containing 0.5 μl of both PCR-products, 1 μl of *Bst*UI (Westburg, Leiden, The Netherlands), 4 μl of the appropriate buffer and 14 μl milliQ water (Millipore, Bellerica, MA, USA), was incubated at 60°C during 3 h. Five μL of the restriction reaction was purified by ethanol precipitation. The obtained pellet was resolved in 13.1 μl deionised formamide (AMRESCO, Solon, Ohio), 0.1 μl ROX500 and 0.3 μl HD400 GeneScan size standards (Applied Biosystems, Foster City, CA) followed by denaturation at 96°C for 2 min and immediate cooling on ice. The restriction fragments were electrophoresed on an ABI PRISM 310 (Applied Biosystems), whereby only the fluorescently labelled 5' terminal restriction fragments (TRFs) were visualized.

The T-RFLP pattern obtained from a sample with a mixed microflora consists of one TRF for each of the different species present. Theoretically the number of peaks (TRFs) reflects the number of different species present in a sample. Identification of the peaks in a T-RFLP pattern, in other words assignation of a species name to each TRF, is based on comparison with a library composed of TRFs that have been obtained from pure cultures of well-identified reference strains or pure 16S rDNA clones, identified by sequence determination. The TRF length of a single species can also be determined by carrying out computer assisted (i.e. virtual) restriction analysis of published 16S rRNA sequences. The peak values in the library entries are the averages of the peak values obtained after testing different strains or cloned 16S rRNA genes of each species.

The choice of the restriction enzyme used is important. We chose *Bst*UI, based on *in silico *analysis of 16S rRNA genes [39] and on literature [40], indicating that this restriction enzyme was well suited for maximal differentiation between *Lactobacillus *species based on the length of the terminal 5' restriction fragment of their 16S rDNA, i.e. their TRF.

T-RFLP patterns were obtained as table files from the Genescan Analysis software and were analyzed using BaseHopper, a software program developed at our university [36]. Using these sample files containing TRF lengths (peak values) in base pairs, this program enabled us to assign a species name to each TRF by comparing each TRF of a T-RFLP fingerprint separately with the library. TRFs with a peak height of less than 10% of the highest peak were excluded from the analysis, since such peaks rarely corresponded with any of the species shown to be present by cloning [41].

### Statistical analysis

To compare rates of occurrence of events, ordinary risk ratios with 95% confidence intervals were calculated, except for paired data, in case of which we calculated McNemar odds ratios with 95% confidence intervals. Statistical significance was accepted at the two-tailed α = 0.05 significance level. All analyses were performed with the statistical software package SPSS version 15.0 (SPSS, Chicago, Illinois).

## Authors' contributions

HV and MT participated in the development of the study design, the collection of the study samples, the collection, analysis and interpretation of the data, and in the writing of the report. RV, GC, EDB and MV participated in the development of the study design, the analysis of the study samples, the collection, analysis and interpretation of the data, and in the writing of the report. All authors read and approved the final manuscript.
